# Architecture and Dynamics of the Wounding-Induced Gene Regulatory Network During the Oolong Tea Manufacturing Process (*Camellia sinensis*)

**DOI:** 10.3389/fpls.2021.788469

**Published:** 2022-01-27

**Authors:** Yucheng Zheng, Qingcai Hu, Yun Yang, Zongjie Wu, Liangyu Wu, Pengjie Wang, Huili Deng, Naixing Ye, Yun Sun

**Affiliations:** ^1^Key Laboratory of Tea Science, College of Horticulture, Fujian Agriculture and Forestry University, Fuzhou, China; ^2^Shenzhen Branch, Guangdong Laboratory for Lingnan Modern Agriculture, Genome Analysis Laboratory of the Ministry of Agriculture, Agricultural Genomics Institute at Shenzhen, Chinese Academy of Agricultural Sciences, Shenzhen, China

**Keywords:** oolong tea, RNA-seq, wounding treatment, volatile, transcriptional reprogramming events

## Abstract

Understanding extensive transcriptional reprogramming events mediated by wounding during the oolong tea manufacturing process is essential for improving oolong tea quality. To improve our comprehension of the architecture of the wounding-induced gene regulatory network, we systematically analyzed the high-resolution transcriptomic and metabolomic data from wounding-treated (after turnover stage) tea leaves at 11 time points over a 220-min period. The results indicated that wounding activates a burst of transcriptional activity within 10 min and that the temporal expression patterns over time could be partitioned into 18 specific clusters with distinct biological processes. The transcription factor (TF) activity linked to the TF binding motif participated in specific biological processes within different clusters. A chronological model of the wounding-induced gene regulatory network provides insight into the dynamic transcriptional regulation event after wounding treatment (the turnover stage). Time series data of wounding-induced volatiles reveal the scientific significance of resting for a while after wounding treatment during the actual manufacturing process of oolong tea. Integrating information-rich expression data with information on volatiles allowed us to identify many high-confidence TFs participating in aroma formation regulation after wounding treatment by using weighted gene co-expression network analysis (WGCNA). Collectively, our research revealed the complexity of the wounding-induced gene regulatory network and described wounding-mediated dynamic transcriptional reprogramming events, serving as a valuable theoretical basis for the quality formation of oolong tea during the post-harvest manufacturing process.

## Introduction

Oolong tea products with rich aromas usually have better economic benefits than those without these aromas. Tea-related practitioners have been working hard to improve the aroma of oolong tea products. Many reports have shown that pre-harvest treatments, such as shading (Yang et al., 2012), different light qualities (Fu et al., 2015), and different seasons (Ji et al., 2018; Xu et al., 2018) and post-harvest treatments, including irradiation (solar withering) (Hu et al., 2018; Yu et al., 2020) and wounding (turnover) (Zhou et al., 2017), could intensify special tea product flavors. In particular, post-harvest treatment is the key step in aroma formation (volatile compounds). Yaoqing (turnover) is a unique post-harvest processing technology used on oolong tea that aims to damage the leaf edge of the tea plant by continuous vibration or shaking (wounding stress) to induce a wide range of metabolic and transcriptional reprogramming events in tea leaves (Chen et al., 2020). Recent studies have shown that the turnover process leads to the accumulation of indole, *E*-nerolidol, and jasmine lactone, which are the representative volatile constituents of oolong tea, by activating the expression of key genes in the related pathway (Baldermann et al., 2014; Zeng et al., 2019b). Interestingly, these three representative substances are synthesized from different pathways, which suggests that the molecular regulatory network driven by wounding stress is comprehensive and complex. During the actual production process, on the other hand, the tea master usually decides the time interval of the turnover stage by experience, which has definite subjectivity. All of the above actions pose challenges to the standardization and refinement of the quality of oolong tea in the future.

Hence, to improve the quality of oolong tea products and their associated processing efficiency, it would be helpful to have a detailed and comprehensive understanding of how tea leaves respond to wounding during the turnover stage. In recent years, the rapid development of omics has provided a new direction for studying the formation mechanism underlying tea quality following post-harvest processing (Huang et al., 2020; Yu et al., 2020; Zhu et al., 2020). Previously, genes with a role in or those showing significantly different expression during the turnover stage have been identified either by some exogenous gene expression system or by using quantitative real-time PCR (qRT-PCR) and transcriptome tools (Zeng et al., 2019b; Zhou et al., 2020). For example, the transcription level of *CsADH* peaks at the turnover stage, and it is involved in the formation of C_6_-alcohols during the oolong post-harvest period (Zhou et al., 2020). The lipoxygenases (LOXs), especially *CsLOX1*, play an important role in the accumulation of jasmine lactone under continuous wounding stress (Zeng et al., 2018). Similarly, the *CsTSB2* and *CsNES* genes were confirmed to be involved in the accumulation of indole and (*E*)-nerolidol during continuous mechanical damage (Zeng et al., 2016; Zhou et al., 2017). Although previous studies have primarily focused on the function of a single gene or transcriptomic and metabolic research at limited time points, much remains unknown about the wounding-induced gene regulatory network.

A detailed understanding of how to adjust the response to wounding treatment during the oolong tea manufacturing process is important to uncovering clues that can improve the quality of oolong tea. Here, we generated a high-resolution time series of the wounding stress-mediated transcriptional response in tea leaves by RNA-seq analysis post-harvest. Several distinct wounding-induced clusters were identified and used to predict some TF families that play an important post-harvest role. A chronology of wounding-mediated transcriptional reprogramming was constructed as well. In addition, a high temporal-resolution volatile metabolome was conducted by gas chromatography-time-of-flight mass spectrometry (GC-TOF-MS). A WGCNA analysis for integrating metabolome and transcriptome data provides insight into the regulatory network of key volatile substance formation. Our study will shed light on the architecture and dynamics of the wounding-induced gene regulatory network and provide insight into the molecular mechanisms underlying key volatile formation, which will provide an important clue to improve the quality of oolong tea.

## Materials and Methods

### Plant Materials and Wounding Treatments

Fresh tea leaves (*Camellia sinensis* var. Jinguanyin) were plucked from the Jinguanyin tea garden on Xuefeng Mountain (Fujian Province, China). The freshly plucked tea leaves were first placed indoors at room temperature for 2 h. Immediately afterward, they were subjected to the turnover process according to previous research with some modifications (Wu et al., 2020). The turnover process (wounding treatment) lasts 10 min in a rotary machine at a rate of 20 r/min and followed by a standing process on bamboo sieves (treatment group: Y-group). We set up a control group (M-group), in which the plucked fresh tea leaves were processed without conducting the turnover process (without wounding treatment) and were just left standing on bamboo sieves. For time-series transcriptomics analysis, the tea leaves were sampled at 11 time points over 220 min for each treatment (Y-group and M-group). For the time-series metabolomics analysis, the tea leaves were sampled at 11 time points over 220 min from the Y-group as well. Three independent biological replicates were collected at each time point and immediately frozen in liquid nitrogen. The detailed processing parameters for the entire experimental design and the sampling time points are shown in Supplementary Figure 1.

### Chemicals

Authentic standards of linalool, benzyl alcohol, *E*-nerolidol, farnesene, phenylethyl alcohol, methyl salicylate, indole, and ocimene were purchased from Sigma Aldrich.

### Wounding-Induced Volatiles Analysis

One gram of the tea sample powder was transferred immediately to a 20 ml headspace vial sealed using crimp-top caps with TFE-silicone headspace septa. There were three biological replicates per sample. At the time of SPME analysis, each vial was placed in 100°C for 5 min, then a 120 μm fiber (Agilent) was exposed to the headspace of the sample for 15 min at 100°C. Wounding-induced volatiles were detected using an Agilent 7890B gas chromatograph (Agilent Co., Santa Clara, CA, United States) coupled with a Pegasus HT time-of-flight mass spectrometer (LECO Co., Saint Joseph, MI, United States) (GC-TOF MS). The identification and quantification of wounding-induced volatiles were performed with a Restek Rxi^®^-5Sil MS capillary column (30 m × 0.25 mm × 0.25 μm film thickness) with a carrier gas (helium gas 99.99%) at a flow rate of 1.5 mL/min. The desorption of the VOCs from the injection port was controlled at 250°C for 5 min in splitless mode. The transfer line temperature was set as 270°C; the following temperature program was used: oven temperature program started at 50°C and maintained for 5 min, increase to 210°C at a rate of 3°C min^–1^, hold for 3 min, then increase to 230°C at a rate of 15°C⋅min^–1^; solvent delay time set as 300 s; mass spectra ion source temperature set at 250°C; and electron energy and detector voltage set at −70 and 1,520 V, respectively. The scan range was set to 30–500 atomic mass units (AMUs) with a 10 spectra/s acquisition rate.

Raw data were processed using the ChromaTOF platform (v4.51.6, LECO, St. Joseph, MI, United States) with a peak width of 5 s and an S/N ratio of 10, auto smoothing, and a baseline offset of 1. The metabolites were identified by matching the retention time and mass spectra with those of the authentic standards and the NIST library. A combined sample [quality control (QC)] prepared by mixing 0.1 g of every sample was used to check the instrument performance.

### High-Throughput RNA-seq

RNA extraction, library construction, and sequencing were performed according to our previous method (Zheng et al., 2021). Total RNA was extracted using TRIzol reagent kit (Invitrogen, Carlsbad, CA, United States) according to the manufacturer’s protocol. An Agilent 2100 Bioanalyzer (Agilent Technologies, Palo Alto, CA, United States) and RNase-free agarose gel electrophoresis were used to assess the total RNA quality. After quality check, eukaryotic mRNA was enriched by Oligo (dT) beads, while prokaryotic mRNA was enriched by removing rRNA by Ribo-Zero™ Magnetic Kit (Epicentre, Madison, WI, United States). The high-quality mRNA was fragmented using fragmentation buffer. Then, the reverse-transcribed cDNA fragments were purified with QiaQuick PCR extraction kit (Qiagen, Venlo, Netherlands), end-repaired, poly (A) added, and ligated to adapters. An Illumina HiSeq2500 platform was used for sequencing. After removing the poly-N, adapter, and low-quality reads, clean data and the Q20, Q30, and GC content values were obtained (Supplementary Dataset 1). The clean reads were uniquely aligned to the latest reference genome (cv. Tieguanyin) (Zhang et al., 2021) using HISAT2.2.4 (Anders et al., 2015) with the default parameters. The expression abundance and variations were estimated in fragments per kilobase of transcript per million fragments mapped (FPKM). Transcriptome data have been uploaded to NCBI database (SRA accession: PRJNA768951 and PRJNA769258).

For RNA differential expression analysis (DEGs), the gene that was significantly differentially expressed after wounding treatment (treatment group: Y-group) compared with the control group (M-group) was identified using DESeq2 (Love et al., 2014). All the genes that did not meet the following requirements were not considered for further analysis: false discovery rate (FDR) < 0.05 and absolute fold change (Y-group vs. G-group, e.g., M-5 vs. Y-5, M-10 vs. Y-10) ≥ 2 at least one of the 10 time points, count number >10 in the lowest expressed sample. Of all the differentially expressed genes, the time point of the first differential expression was predicted.

### Principal Component Analysis

Principal component analysis (PCA) was performed with two R packages, FactoMineR (for the data analysis) and Factoextra (for the data visualization based on ggplot2), on the gene expression profiles (Irnawati et al., 2021).

### Functional Enrichment Analysis

Gene function was annotated in the Gene Ontology (GO) databases and Kyoto Encyclopedia of Genes and Genomes (KEGG) databases using the BLASTX algorithm (*E*-value ≤ 1e−5) to obtain the KO and GO numbers. The enrichment analysis was conducted using the ClusterProfiler v4.0.0 R package (Yu et al., 2012). Overrepresentation of the GO terms and KEGG pathways was identified by calculating a *p*-value (hypergeometric distribution, *p* ≤ 0.05).

### Analysis of Gene Expression Pattern

An analysis of differentially expressed genes was performed using the Mfuzz R package (Kumar and Futschik, 2007) on a data frame of the fold changes (Y-group vs. M-group) at each time point with a default standardized procedure. Other parameters were maintained as defaults.

### Transcription Factor Annotation and Promoter Motif Analyses

All the genes were annotated in the Plant transcription factor (TF) database (PlantTFDB v5.0)^1^ to determine whether they are TFs. Overrepresentation for the TF families was recognized by calculating a *p*-value (hypergeometric distribution, *p* ≤ 0.05) using the Phyper function in R studio. TF-corresponding motifs 500 bp upstream of the promoter sequences for all the genes in a given cluster were scanned by PlantPAN using an *Arabidopsis thaliana* TF position weight matrix (Chow et al., 2019). Overrepresentation of the TF-corresponding motifs was identified by calculating a *p*-value using the Phyper function in R studio. *De novo* motifs were discovered by using MEME STREME v5.3.3 algorithms, which were run using a 2nd-order Markov model with the following parameter settings: streme -verbosity 1 -minw 5 -maxw 15 -pvt 0.05 (Fisher’s exact test, *p* < 0.05).

### Identification of Chronological Phases

According to a previous method (Hickman et al., 2017), we first classified all DEGs according to whether they were either up- or down-regulated in response to wounding and then further determined whether they were either TFs or structural genes. This screening method identified four mutually exclusive sets of wounding-induced genes (i.e., an up-regulated gene set and a down-regulated TF set). Next, the expression matrix of the gene set was used to calculate the correlation between all pairs of time points with the Pearson correlation coefficient. Then, each resulting correlation matrix was subjected to column clustering using the Euclidean distance. The clustering results were used to infer specific phases of wounding-induced transcription. One gene in the four gene sets is assigned to the transcription phase according to its time point of first differential expression.

### Weighted Gene Co-Expression Network Analysis

The WGCNA package was used to build a co-expression network (Langfelder and Horvath, 2008). The matrix of all the FPKM values of the DEGs at each time point was standardized and transformed into an adjacency matrix using the default formula (connection strength = | (correlation + 1)/2| ^β^). The β value indicates the soft threshold of this correlation matrix, which gives a higher weight value to the strongest correlations. The PickSoftThreshold command was used to determine the best soft threshold and the corresponding average connectivity, and the results showed that a β value equal to 16 was the best soft threshold (Supplementary Figure 2). The blockwiseModules command was used for gene network construction and module identification with the following parameter settings: mergeCutHeight = 0.25, minModuleSize = 30, and other parameters were kept in the defaults. To determine the modules most relevant to some representative volatile substances, we calculated the module eigengene values (ME value) of each module using the moduleEigengenes command and then calculated the correlation between the representative volatile components and the ME values of each module. A positive correlation indicated that the genes in a module may have a potential relationship with volatile components. The TF regulatory network and KEGG pathway network of some important modules were constructed using Cytoscape v3.8.2 (Shannon et al., 2003).

### Quantitative Real-Time-PCR Analysis

To validate the accuracy of our high-resolution transcriptome data, 10 candidate genes were selected. An RNAprep Pure Plant Kit (Tiangen, Beijing, China) was used to extract the RNA templates. RNA was reverse-transcribed using cDNA Synthesis SuperMix (TransGen, Beijing, China). PCRs were performed in 96-well plates with a CFX96™ real-time PCR system (Bio-Rad, United States) using the TransStart Tip Green qPCR superMix kit (TransGen). All the specific primers were designed on the primer3plus website using default parameters, which are listed in Supplementary Dataset 3. The *CsGAPDH* gene was chosen as a reference for normalization. The relative gene expression levels were measured using the 2^–ΔΔCt^ method, and the verification results are listed in Supplementary Figure 3.

## Results

### Overview of a Time Course of Metabolome and Transcriptome Data

A time course of the transcriptome and metabolome analysis can provide comprehensive insight into different levels of dynamic reprogramming events that take place in tea leaves following wounding stimulation during the oolong post-harvest period. In this study, RNA-seq strategies were used to obtain the genome-wide transcript level in tea leaves before wounding (turnover stage) and over 10 consecutive time points within 220 min following the stimulation of wounding stress or untreated tea leaves (control group). The tea leaves at each time point that yielded 63 samples (three independent biological replicates) were used for total RNA isolation. The process for RNA extraction, library construction, and bioinformatics analysis is listed in the “Materials and Methods” section. To summarize, a total of 15690 genes showing significant differential expression for at least one time point were identified between wounded and untreated tea leaves.

**FIGURE 1:**
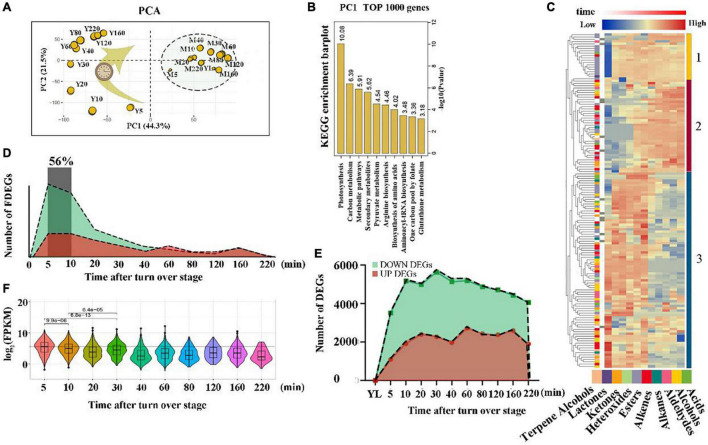
Overview of a time course of metabolome and transcriptome data. **(A)** RNA-seq score plot of a principal component analysis (PCA). The size of the yellow circle represents their contribution values to the principal components. **(B)** KEGG enrichment analysis of the top 1000 genes in PC 1 (the most 1000 differential expressed genes between all wounding time points vs. all non-wounding time points). Yellow columns indicate the *p*-value of the top 10 significantly enriched pathways. **(C)** Heat map of metabolic profiles. The peak value of each metabolite was normalized [log2 (peak area + 1)] to complete the linkage hierarchical clustering. Red indicates high abundance. The different color blocks on the left side of the heat map represent different types of wounding-induced volatiles; the different color blocks and number on the right represent different clusters. **(D)** The number of FDEGs of an area chart. FDEGs: The gene was differentially expressed for the first time at a specific time point. Green indicates down-regulated genes, whereas red indicates up-regulated genes. **(E)** The number of DEGs in an area chart. Green indicates down-regulated genes, whereas red indicates up-regulated genes. **(F)** Violin plot of FDEG expression distribution at each time point. The expression of each FDEG was normalized using log2 (FPKM).

To explore global differences in the transcriptome dynamics of tea leaves in response to wounding stress post-harvest, a PCA was conducted on all the expressed genes (Figure 1A). The PCA results showed that biological replicates clustered closely, demonstrating that synchrony was maintained and that the tea samples after wounding stress (Y-Group) were distributed regularly along the time trajectory, indicating that time was the major contributor to explaining the variance and that the samples between adjacent time points had more similar global expression patterns. The untreated tea sample group (M-Group) closely clustered together. The above results indicated that wounding stress induced dramatic (changes rapidly in a short time) transcription reprogramming events in tea leaves. In addition, the first dimension (Dim 1) separates the M-Group and Y-Group effectively, indicating that a trend in the wounding treatment effect on the transcription level is visible. The top 1000 genes that had the best representation of the variable on the principal component (Dim 1) were primarily enriched in the photosynthesis pathway and some primary and secondary metabolite pathways of the tea leaves (Figure 1B), implying that wounding stress during the oolong tea post-harvest primarily affected the changes in tea plant metabolites and photosynthesis.

Next, we examined the number of DEGs (Figure 1E) between the Y-Group and M-Group and the number of differentially expressed genes appearing for the first time between the two groups (FDEGs) (Figure 1D). The results showed that the number of DEGs between the Y-Group and M-Group was within a small variation range at different time points. The number of FDEGs followed a clear single-pulse pattern over time, which peaked significantly within 5–10 min after stimulation and accounted for 56% of the total number of DEGs. Furthermore, we checked the distribution of gene expression for FDEGs at each time point (Figure 1F). The analyses showed that the expression of gene clusters (5 and 10 min) was significantly higher than that of other time point clusters. In conclusion, our results captured a global burst of wounding-induced gene transcription programs generally within 10 min after treatment, indicating that tea leaves respond rapidly to external damage stress signals and mediate a relatively short cascade reaction.

A trend in the wounding treatment effect on the metabolite level is apparent as well. The results of the heat map visually showed the dynamic trend in volatile metabolites after wounding stress during oolong tea post-harvest (Figure 1C). These volatile metabolites were primarily divided into three groups. A deep insight into those volatile metabolites within Cluster 1 showed that most of them were esters with a special accumulation pattern: the accumulation level remained relatively consistent after wounding stress within 220 min, while at the YL stage (without wounding stress), the content was relatively low or undetected. The volatile metabolites in Cluster 2 began to accumulate significantly 40 min after wounding treatment. Notably, indole, farnesene, *E*-nerolidol, and ocimene, the most important aroma components of oolong tea (Lin et al., 2013; Yang et al., 2013; Baldermann et al., 2014), were clustered in Cluster 2. In contrast to the accumulation pattern of cluster 1 and cluster 2, the volatile metabolites in cluster 3 including hexanal, (*Z*)-3-hexenal, (*Z*)-3-hexen-1-ol, *cis*-3-hexenyl isovalerate, (*Z*)-2-penten-1-ol, (*E*)-2-non-enal, and 1-heptanol were abundant in fresh leaves (YL stage) and began to decrease approximately 40 min after the wounding treatment (Supplementary Figure 4 and Supplementary Dataset 2). Our results suggest that most of the substances with a grass odor such as (Z)-3-hexen-1-ol (Wang et al., 2017), 1-heptanol (Wang et al., 2017), and hexanal (Wen et al., 2014) gradually disappear within 40 min after wounding treatment, while some substances with flower and fruit flavors gradually accumulate after 40 min of wounding treatment, which may be the potential reason why it is necessary to keep tea leaves motionless after wounding treatment for a while during oolong tea possessing (Gui et al., 2015; Zeng et al., 2019a; Chen et al., 2020).

### Expression Pattern-Specific Gene Clusters

**FIGURE 2:**
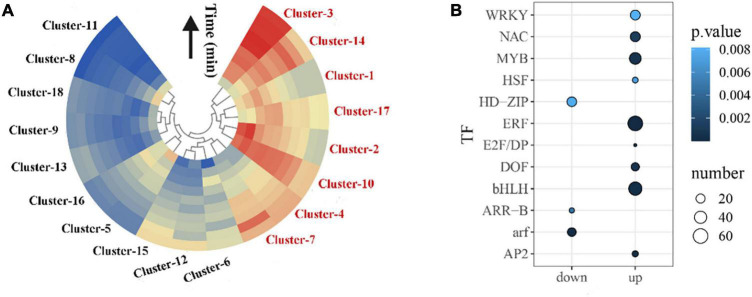
Clustering of co-expressed genes in the wounding response gene regulatory network. **(A)** A total of 18 distinct clusters that share similar expression dynamics were yielded using the Mfuzz package in R. The mean gene expression profile for each cluster was visualized with a heat map. Red and blue indicate up- and down-regulation of expression [log2 (fold change)]. Fold change = Y-Group/M-Group (e.g., Y-5/M-5). **(B)** Significantly overrepresented TF families in all clusters t (hypergeometric test; *p* < 0.05).

To investigate the specific expression pattern clusters during oolong tea post-harvest, a time series clustering analysis was conducted using the Mfuzz package. A total of 18 distinct clusters were yielded, which were generally divided into two major groups: those that displayed increased expression in response to wounding stress during oolong tea post-harvest (clusters 1–4, 7, 10, 14, and 17) and those that showed reduced expression in response to wounding stress during oolong tea post-harvest (clusters 5, 6, 8, 9, 11–13, 15, 16, and 18) (Figure 2A and Supplementary Figure 5). Our results showed that most of the clusters activated by wounding stress post-harvest possess a clear pulse-like expression pattern. Some clusters shared rapidly up- or down-regulated induction in response to wounding stress, broadly starting within 5 min and peaking within 20 min, such as clusters 2 and 12. Some clusters showed largely sustained induction, such as clusters 8 and 15 (Supplementary Figure 5).

Plant TFs play an important role in the response to environmental stress, and they are also key switches in transcriptional regulatory networks. We found that the bHLH, ERF, NAC, MYB, WRKY, HSF, E2F/DP, DOF, and AP2 TF families were most significantly overrepresented (*p* < 0.05) within up-regulated clusters and HD-ZIP, ARR-B, and ARF TF families were significantly overrepresented within down-regulated clusters, indicating that these TF families may play a dominant role in regulating the series of wounding-induced gene expression (Figure 2B).

### Overrepresentation of Transcription Factor Binding Motifs

**FIGURE 3:**
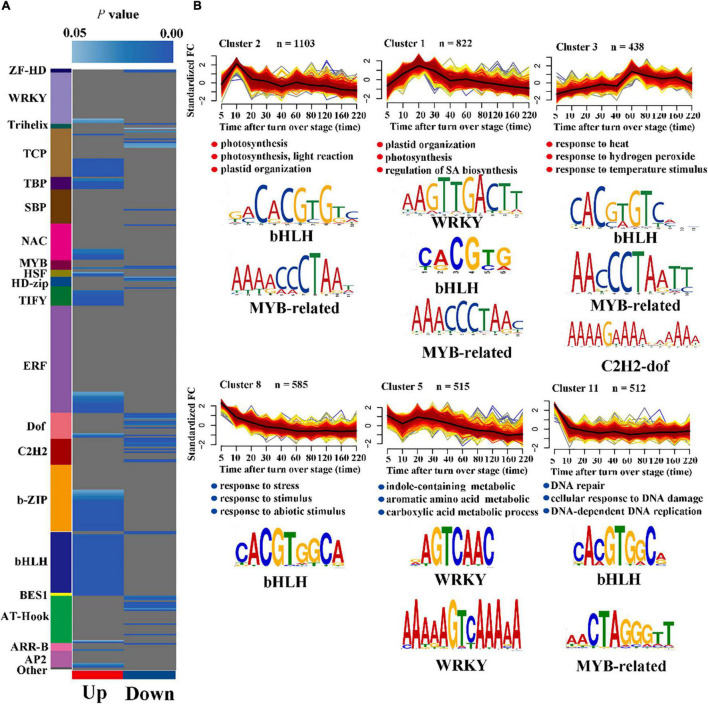
Enriched TF corresponding motifs and GO enrichment analysis in a wounding-responsive cluster. **(A)** The overrepresentation of TF binding motif within the up- and down-regulated cluster. Different color blocks represent different transcription factor families. Blue indicates a motif that is significantly overrepresented in the up- or down-cluster (cumulative hypergeometric distribution). **(B)** Typical co-expression clusters (line chart) with their newly discovered (*de novo*) TF-corresponding motifs (*p* < 0.01, cumulative hypergeometric distribution). The blue dots represent the significantly enriched GO terms (full GO results are available in Supplementary Dataset 4).

Transcription factors regulate the precise initiation and transcription efficiency of gene transcription by specific binding with *cis*-regulatory elements. Studying the TF-binding site contained in promoters of gene sets with specific expression patterns aids in understanding wounding response gene regulatory networks during the post-harvest period for oolong tea. First, we investigated the overrepresentation of TF-corresponding motifs in both up- and down-regulated clusters using Plant Promoter Analysis Navigator 3.0 with a TF position weight matrix (Figure 3A). The binding sites of WRKY, TBP, NAC, TIFY, ERF, b-ZIP, and bHLH were significantly overrepresented in the up-regulated clusters, while the motifs of HD-zip, Dof, and C2H2 were markedly overrepresented in the down-regulated clusters. Combined with the TFs that were significantly overrepresented within the up- and down-regulated clusters, our results further demonstrated that the bHLH, ERF, WRKY, NAC, and HD-zip TF families dominated the series of wounding-induced gene expression during the post-harvest period for oolong tea.

Next, we conducted *de novo* motif discovery analysis to discover TF binding sites in six clusters with typical co-expression patterns to discover the further potential relationship between TFs and gene sets with similar expression patterns. Our results showed that motifs were selectively overrepresented in typical clusters, indicating that the different TFs may be involved in different biological processes (Figure 3B and Supplementary Dataset 4). The WRKY TF family is known as an important regulator of the SA response pathway. Cluster 1, which was enriched for the SA-related pathway, significantly enriched the WRKY-corresponding motif, suggesting that WRKYs are related to the regulation of salicylic acid in response to wounding stress. In addition, only WRKY-corresponding motifs were significantly enriched in cluster 5, indicating that the WRKY TFs may also be associated with some secondary metabolite pathways, such as indole-containing metabolic and aromatic amino acids. Cluster 8 was enriched for GO terms describing stress response pathways, implying that bHLH TFs play an important role in the early response to wounding stress.

### Chronology of the Wounding-Triggered Gene Regulatory Network

High-resolution time-series transcriptional information was used to construct the chronology of the wounding-triggered gene regulatory network. First, we divided the DEGs into two groups with up- and down-regulated expression levels and then separated them into two additional groups according to their predicted function (as a TF or as regulated gene). Discovering the key time point determining the coordinated switch in transcriptional activity is helpful to understanding the transcriptional reprogramming events of tea leaves in response to wounding stress. Generally, two adjacent time points with a weak correlation are the key time points, which indicate the important turning point and coordinated switches in transcription reprogramming events.

**FIGURE 4:**
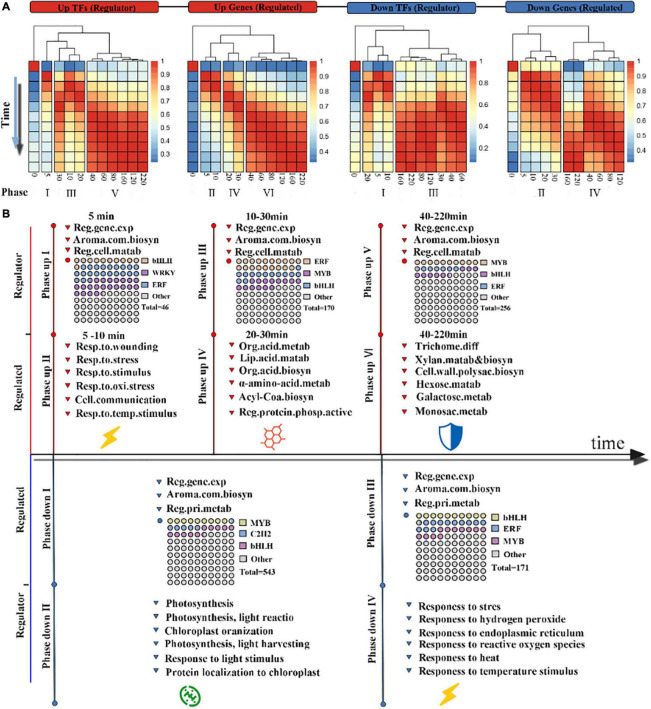
Chronology analysis of the wounding-induced gene regulatory network: **(A)** various phases of wounding treatment induction. Differentially expressed genes were divided into four categories based on their annotation as a transcription factor or a structural gene and their expression patterns. The correlation matrix of gene expression between all pairs of time points was calculated by Pearson correlation and was further subjected to column clustering. Red and blue indicate high and low correlations, respectively. The time is in min. **(B)** Chronology chart of the wounding-induced gene regulatory network. The above part of the timeline represents the up-regulated phase, and the bottom part represents the down-regulated phase. The FDEGs in each phase were subjected to GO enrichment analysis. The waffle diagram shows the top 3 TF families in the gene set.

Therefore, we calculated the correlation of transcription levels between all adjacent time points in each group and obtained the correlation matrix of each group. A cluster analysis was performed on the columns of each correlation matrix, and the results showed that six phases in transcriptional activation and four phases in transcriptional repression were partitioned (Figure 4A). For the up-regulated regulators, the first phase of the Up-TFs group started within 0.5 min after wounding stress, while at 10 min, phase up III of the Up-TFs group ensued. Then, phase up V of the Up-TF group started 40 min after wounding stress. For the up-regulated genes, the phase up II and phase up VI start at the same time as the phase up I and phase up V of up-regulated regulators. The phase up IV of up-regulated genes was later than the phase up III of the regulatory genes. Similar transcriptional regulatory events could be observed in the two down-regulated groups. The result of confirming the key time points determining the coordinated switch may indicate that tea leaves showed a complex and rapid transcriptional regulation mode in response to wounding stress, that is, it follows the fast-slow-fast transcriptional regulation mode within 200 min.

The transcriptional regulation of key biological processes is an important feature in investigating the complexity and directionality of the wounding stress response process. The phase up I and II in up-regulated genes and regulators represents the rapid transcriptional response. Genes encoding ERFs, bHLHs, and WRKYs were the most abundant TFs in phase up I, suggesting that they play an important role in the early response to wounding stress (Figure 4B and Supplementary Dataset 5). In addition, genes in phase up II are primarily associated with the response to stress, such as the response to wounding, implying that this process is one of the first targets of wounding-mediated up-regulated transcriptional events. In the next two phases (III and V), we found that bHLHs and ERFs were still the two TFs appearing in the highest proportions, which emphasized the importance of these two TFs in response to wounding stress during the oolong tea process (Figure 4B). Strikingly, the number of MYB TFs increased gradually in the above two phases. The two phases corresponding to the above phrases were found to involve completely different biological processes. Phase up IV primarily involves the synthesis and metabolism of secondary metabolites, such as fatty acid metabolism and organic acid biosynthesis; phase up VI is primarily involved in the primary metabolism of plants, such as monosaccharide and galactose metabolic processes. Overall, wounding-mediated transcriptional reprogramming events showed an obvious chronology of up-regulated genes, which started with the activation of wounding stress-related pathways, followed by the activation of defense-related secondary and primary metabolic pathways.

Repressed transcriptional events are divided into four phases: phase down I, phase down II, phase down III, and IV, which start at 5, 5, 30, and 40 min after wounding treatment, respectively (Figure 4B). An interesting phenomenon is that having a large number of repressed genes in the early phase emphasizes that the wounding stress signal inhibits the photosynthesis of tea leaves in phase down II (Supplementary Dataset 5). The genes that encode C2H2 and other TFs in phase down I may be related to this inhibition process. Interestingly, the genes in the later phase (phase down IV and V) are primarily related to the response to stress, which is similar to the pathway in phase up II. In general, our chronological analysis of the wounding-triggered gene regulatory network revealed the defense strategies of tea leaves when suffering from wounding stress, which is intended to balance the flow of energy between defense and development.

### Transcription Regulatory Modules Related to Key Aroma Compound Formation

**FIGURE 5:**
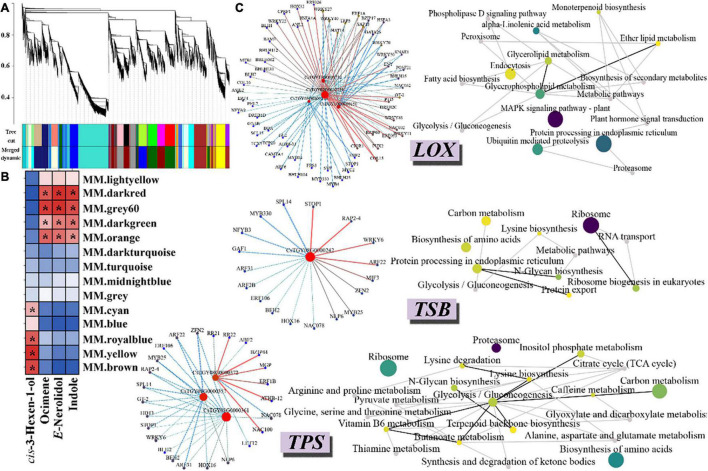
Co-expression network of wounding-induced volatile formation. **(A)** Hierarchical clustering tree of DEGs based on WGCNA analysis. Each short black line (“leaf”) represents an individual gene. Different colored boxes below the hierarchical clustering tree represent genes with similar expression patterns, and these genes were clustered based on dissimilarity measures. Cut tree represents the cut height of the cluster tree; Merged dynamic represents further merge similar modules according to module eigengene. **(B)** Correlation heat map of four volatile substances and each module. Red and blue indicate high and low correlations, respectively. “*” indicates a significant correlation between the module and volatile substances (*p* < 0.05). The correlation value has been scaled. **(C)** Co-expression network diagram of candidate genes. The left side shows the TF-gene regulation network. When the weight value is greater than 0.5, we believe that there is a regulatory relationship between the candidate genes and TFs. The red dots represent the LOX, TSB, and TPS, respectively. The left side shows the KEGG undirected network diagram. The structural genes that have a potential relationship with candidate genes (weight value < 0.15) were chosen to construct a KEGG undirected network. The node size and color represents the number of genes enriched into the pathway. The larger purple node represents more genes in this pathway, and smaller yellow circles represent fewer genes.

To investigate the gene regulatory network of aroma formation comprehensively after wounding stress, a total of 11203 DEGs were used to conduct a weighted gene co-expression network analysis (WGCNA). After the merging of similar modules, 13 modules were generated, which comprised 153–2776 genes (Figure 5A). All the modules contain TF-encoding genes, and the TF-encoding genes in some modules have very high connectivity, such as *WRKY27* and *48* in the brown module and *MYB20* in the dark red modules, revealing the tight regulation and complexity of the transcriptional activity. Indole, *E*-nerolidol, ocimene, and *cis*-3-hexen-1-ol, the most important aroma components of oolong tea, have been reported to be synthesized in three different biological pathways. Therefore, to reveal the complex transcriptional regulation network of aroma formation after wounding treatment during the oolong tea process, the correlation between the above four representative aroma components and each module was calculated by using the peak area data at each time point after wounding treatment. The results showed that the peak area of *cis*-3-hexen-1-ol at each time point after wounding treatment was positively correlated with the cyan, blue, royal blue, yellow, and brown modules, while the indole, *E*-nerolidol, and ocimene modules were positively correlated with the dark red, grey60, dark green, and orange modules, indicating that these modules play an important role in aroma formation during the oolong tea process (Figure 5B).

Further analysis showed that three lipoxygenases (CsTGY13G0001710, CsTGY10G0000150, and CsTGY02G0002259) and one lipoxygenase (CsTGY03G0001270) were present in the gray module and the cyan module, respectively. There may be a strong relationship between the two nodes when the weight value is greater than 0.15. Therefore, we screened out TF-encoding genes and genes that directly connect with the above four *LOX* genes (weight value greater than 0.15) in the gray and cyan modules to construct the transcriptional regulatory network and to conduct KEGG enrichment analysis. The results showed that there were 30 different TF families that may be involved in directly or indirectly regulated *LOX* gene expression. Among them, *bHLH35* (CsTGY11G0000029) and *DREB1D* (CsTGY11G0000597) showed the strongest relationship to *LOX* (CsTGY03G0001270); *CO-like* (CsTGY06G0000080) and *B3* (CsTGY08G0000618) showed the strongest relationship to *LOX* (CsTGY02G0002259); *GRAS* (CsTGY05G0000463) and *CO-like* (CsTGY06G0000080) showed the strongest relationship to *LOX* (CsTGY13G0001710); and *WRKY27* (CsTGY14G0001458) and *C2H2* (CsTGY10G0002372) showed the strongest relationship to *LOX* (CsTGY10G0000150), indicating that these TFs play an important role in regulating the expression of *LOX* and the formation of *cis*-3-hexen-1-ol during oolong tea processing. In addition, a large number of *LOX*-related genes were significantly enriched in the MAPK signaling pathway, indicating that the MAPK cascade pathway plays an important role in the response to wounding stress and mediates the formation of *cis*-3-hexen-1-ol (Figure 5C).

We observed that the tryptophan synthase β-subunit (*CsTSB*: CsTGY08G0000242), which directly participates in indole biosynthesis, was located in the dark green module. The two TFs *C2H2* (CsTGY04G0001773) and *ERF* (CsTGY04G0000903) have the strongest regulatory relationship with *CsTSB*. KEGG enrichment analysis showed that some pathways participated together in indole biosynthesis, such as the lysine biosynthesis pathway and N-glycan biosynthesis pathway. In addition, three terpenoid synthetases, including one ocimene (*CsOCS*: CsTGY08G0000372) and two nerolidol synthases (*CsNES*: CsTGY08G0000357 and CsTGY08G0000361), were located in the grey60 module and dark green module, respectively. Twenty-five TFs from 18 different TF families showed a potential regulatory relationship with these three *CsTPSs*. The results of the KEGG enrichment analysis showed that some connected or independent biological pathways were involved in the formation of terpenoids during the processing of oolong tea, such as terpenoid backbone biosynthesis, glycolysis/gluconeogenesis, and the N-glycan biosynthesis pathway (Figure 5C).

Taken together, these findings suggest that the mechanism of aroma formation is complex after wounding treatment and involves many different TF families and a large number of interconnected or independent biological pathways. Our information-rich wounding-induced data help in exploring the mechanistic function of individual TFs and the mechanistic formation of important aroma substances, aiding in a holistic acquaintance with the wounding-induced regulatory network during oolong tea processing.

## Discussion

The comprehensive transcriptional reprogramming events mediated by wounding treatment are poorly understood within the post-harvest process of oolong tea. Our information-rich wounding-induced data enable us to provide new insight into the dynamics and architecture of the wounding-induced gene regulatory network and the regulatory mechanism of aroma formation during the post-harvest processing of oolong tea. Previous studies on the oolong tea manufacturing process attempted to reveal the molecular mechanism of aroma formation in oolong tea processing by using transcriptomic or metabolic strategies with a limited number of time points or by using an exogenous expression system. Our high-density time series data provide a comprehensive description of a wounding-induced burst of transcriptional events that produces a series of expression patterns with specific dynamic characteristics. We also present a detailed survey of the dynamic characteristics of the aroma following wounding treatment. This approach will provide a scientific basis for researchers to unravel the complexity of transcription activity induced by wounding stress during the oolong tea manufacturing process and to improve the quality of oolong tea products.

### The Necessity of Allowing Tea Leaves to Rest for a While After the Turnover Stage

Many characteristic aroma compounds from different biosynthetic pathways are formed after the turnover stage; however, not all volatile substances contribute to the formation of the oolong tea aroma quality, such as some volatile substances with a “fresh green” odor (ul Hassan et al., 2015). Many studies have shown that green leaf volatiles (GLVs) with a “fresh green” odor accumulate in large quantities after the turnover stage, such as (*Z*)-3-hexen-1-ol, hexanal, (*E*)-2-hexen-1-ol, and 1-hexanol (Zhou et al., 2020; Guo et al., 2021). Our rich time series data capture more detailed information about the accumulation pattern of GLVs in tea leaves immediately; that is, the turnover stage can induce a significant accumulation of GLVs, such as (*E*)-2-hexen-1-ol, 1-hexanol, (*Z*)-3-hexen-1-ol, (*Z*)-3-hexenal, and hexanal, but they start to decrease gradually over time, approximately 40 min after a wounding stress, which may be closely related to *CsLOX* following a clear single pulse expression pattern (peaking at 5 min after wounding stress) (He et al., 2020; Figure 1A and Supplementary Figures 3,4). In addition, we also observed that the contents of some substances with a “fresh green” odor, such as *cis*-3-hexenyl isovalerate, (*Z*)-2-penten-1-ol, (*E*)-2-non-enal, and 1-heptanol, had a similar accumulation pattern. More strikingly, the contents of indole, α-farnesene, ocimene, and *E*-nerolidol which are the most important aroma compounds in oolong tea, increased explosively after a while (approximately 40 min after treatment) (Figure 1C and Supplementary Figure 4). In fact, it is necessary to keep tea leaves motionless after wounding treatment for a while in rotary machine. This process is called “Zuoqing” or “Making green.” Our results highlight the necessity of allowing the leaves to rest for a while after the turnover stage, during the actual oolong tea manufacturing process, which is helpful in the formation of the elegant aroma of oolong tea and the reduction of the green odor substances in the tea. Moreover, our results emphasized the concept that adequate temporal resolution is indispensable for exploring biological transitions, and end-point analysis may not completely describe the complex biology of phenotypic change.

### Temporal Dynamics of Gene Transcription in Response to Wounding Stress

The change in gene expression is apparent and rapid across a broad range of timescales when organisms face stimulation and adapt to perturbations (i.e., min to hours) (López-Maury et al., 2008). One interesting finding from our research is that the majority of FDEGs with more intense expression appeared within the first 10 min after wounding treatment, indicating a rapid transcription burst strategy on a min scale to cope with wounding stress during the turnover stage. Consistent with this observation, published studies showed that a similar transcriptional burst event occurred when plants respond to hormone stress, pathogen infection, and waterlogging, indicating that this response may be a conservative defense strategy for plants. Strikingly, more wounding-induced, highly ordered expression patterns that are not limited to the immediate wave of transcription were discovered, such as single-pulse (impulse) patterns (Clusters 1, 2, 3, and 7), sustained patterns (Clusters 4, 8, 11, and 15), and oscillating patterns (Clusters 6 and 10) (Supplementary Figure 5). Single-pulse (impulse) patterns usually interact with each other and form an orderly and multi-step transcription cascade, in which the products of early induced genes activate the expression of downstream targets. In turn, downstream targets may exhibit a variety of expression patterns that trigger a long-term change in the cell state (Chechik and Koller, 2009; Yosef and Regev, 2011). These highly coordinated genes usually help to coordinate the biosynthesis of proteins that are necessary to perform the corresponding cellular functions in response to a given stress. Our results showed that co-expressed gene sets with different expression patterns involve completely different biological functions, which implies that sophisticated regulatory patterns help tea plant leaves respond to wounding stress. Moreover, impulse-like expression changes are closely related to many TFs that play a critical role in promoting the early differentiation of programs (Hickman et al., 2017, 2019). Our results showed that bHLH- and MYB-related TFs may play a leading role in the formation of single-pulse (impulse) patterns or sustained patterns.

In summary, our transcriptome data showed that short transcriptional cascades controlled or led wounding-induced gene activation or repression and generated unique transcriptional signatures that corresponded to specific biological processes after the turnover stage.

### Insight Into the Chronological Model in Response to Wounding Stress

Our gene chronology analysis reflects a particular order of transcriptional reprogramming events. Many genes and TFs that show significant transcriptional changes extremely early, i.e., those in phases I and II are related to the response to wounding. Interestingly, genes involved in responsiveness to other stresses, such as oxidative stress, heat, or chemical stimuli, are strongly induced at phase up II (Supplementary Dataset 5). Previous research reported that the reactive oxygen species (ROS) in damaged tissue production reaches a maximum within a short time after wounding (Vega-Muñoz et al., 2020), which may be the reason why many genes that respond to oxidative stress are enriched in phase up II. Additionally, the massive accumulation of ROS will induce the destruction of the photosystems and trigger photoinhibition (Takahashi and Murata, 2008), which may cause many genes related to photosynthesis to be suppressed in phase down II. Moreover, previous studies have reported that heat stress-related genes could induce somatic embryogenesis (Fehér, 2015). We speculated that the genes involved in heat stimulus in phase up II were associated with repairing damaged plant tissue (León et al., 2001). By contrast, genes in phase up IV that respond transcriptionally later on represent the modulation of primary metabolism, e.g., acetyl-CoA biosynthetic and metabolic processes, lipid metabolism, and carbohydrate metabolism. Acetyl-CoA is a product of the second stage of respiration as well as a raw material in the synthesis of fatty acids, carotenoids and terpenoids. Therefore, we reasonably speculate that phase up IV can not only provide energy to resist external stresses but also provide synthetic raw materials for some important secondary metabolites. In phase up VI, some pathways involved in plant structural defense were significantly enriched, such as “trichome differentiation,” “xylan biosynthetic process,” and “cell wall polysaccharide biosynthetic process.” Our time-course analysis showed that wounding induces dynamic transcription reprogramming events after the turnover stage, starting from rapid wounding-induced responses and photoinhibition followed by the activation of primary metabolic processes and the subsequent activation of structural defense.

### Transcription Factors Play an Important Role in the Response to Wounding Stress During the Oolong Tea Manufacturing Process

Our analysis showed that bHLH TFs appear to be the master regulators controlling most of the wounding-induced genes, while *ERF*, *WRKY*, *MYB*, *NAC*, and *Dof* TFs activate the majority of the wounding-induced target genes, and some of them fine-tune the transcription level of specific sets of target genes in a different cluster. Previous studies have found that *bHLH*, *ERF*, *WRKY*, and *NAC* TFs encode the proteins primarily involved in hormone signal transduction pathways and play a key role in steering secondary metabolite biosynthesis in response to wounding stress (Cheong et al., 2002; De Geyter et al., 2012). For instance, *NAC071* and *ERF113* play an important role in wound-induced auxin accumulation (Asahina et al., 2011), while *WRKY70* is a key node in the SA and JA signaling pathways (Li et al., 2004). We inferred that the various hormones and their respective crosstalk are essential for wounding perception after the turnover stage. While some TFs are not regulated by hormone signals in response to wounding stress. In *Nicotiana attenuata*, *TaWRKY3* is up-regulated in wounding treatment, yet is not activated by treatment with JA (Skibbe et al., 2008). At the same time, previous studies have found that *ERF* could coordinate wounding signaling with the initiations of wound repair mechanisms (Heyman et al., 2018). This implies that TFs play multiple roles in plant defenses in response to wounding stress. Next, we identified a series of TFs that indirectly or directly regulated key aroma substance biosynthesis after the turnover stage by using WGCNA. The roles of some TF families that we identified in this study, such as WRKY, ERF, NAC, MYB, and b-ZIP are well known to regulate the biosynthesis of terpenoids (Xu et al., 2019). In *Dendrobium officinale*, bHLH4 is bound to the G-box element in the promoter of *TPS10* to induce gene expression (Yu et al., 2021). In rice, four key structural genes in MEP pathway are regulated by OsMYC2 (Kamolsukyunyong et al., 2013). Our information-rich wounding-induced data also identified many TFs with high confidence (weight value) that were involved in the synthesis of indole and *cis*-3-hexen-1-ol during the oolong tea manufacturing process.

In summary, this paper provides novel insight into the architecture and dynamics of the wounding-induced gene regulatory network after the turnover stage during the oolong tea manufacturing process by computationally analyzing high-resolution transcriptomic and metabolomic data. Our results reveal the potential scientific significance of some production steps, and it advances our understanding of the actual oolong tea process. Moreover, based on our research, we propose some ideas for optimizing the actual processing of oolong tea. First, reducing the inhibition of photosynthesis by ROS after the turnover stage (phase down II) and providing proper supplementation with light may contribute to the synthesis of the “source,” which is the substrate for the synthesis of various secondary metabolites. Second, the intensity of respiration by tea leaves was reduced after the turnover stage (phase up IV), which may be conducive to the slow consumption of resources to extend the rest time of tea leaves. The above results emphasize the scientific basis and practicality of our research. In the future, we will use ATAC-seq, ChIP-seq, and Y1H technologies to perform functional studies on the TFs screened by WGCNA.

### Supporting Information

Flow chart of the entire experimental design (Supplementary Figure 1); sample photos of each time point (Supplementary Figure 2); the best soft threshold of WGCNA (Supplementary Figure 3); the color information of different modules (Supplementary Figure 4); correlation analysis between RNA-seq of selected genes (Supplementary Figure 5); the important volatile compounds after wounding treatment of oolong tea (Supplementary Figure 6); 18 profiles of log2-fold change in gene expression (Y-group vs. M-group) (Supplementary Figure 7) (PDF); total ion current of volatile metabolites profiling of each sample (Supplementary Figure 8); RNA-seq data quality information (Supplementary Dataset 1); the primers of selected genes (Supplementary Dataset 2); variation and information of metabolic profiling by GC-TOF-MS (Supplementary Dataset 3); GO enrichment analysis of six representative clusters (Supplementary Dataset 4); GO enrichment analysis of each phase (Supplementary Dataset 5) (XLSX).

## Data Availability Statement

The datasets presented in this study can be found in online repositories. The names of the repository/repositories and accession number(s) can be found below: NCBI SRA, PRJNA768951 and PRJNA769258.

## Author Contributions

NY, YS, and YZ conceived and designed the research. YZ, QH, YY, ZW, PW, and HD performed the experiments. YZ and LW analyzed the data. YZ wrote the manuscript. All authors read and approved the manuscript.

## Conflict of Interest

The authors declare that the research was conducted in the absence of any commercial or financial relationships that could be construed as a potential conflict of interest.

## Publisher’s Note

All claims expressed in this article are solely those of the authors and do not necessarily represent those of their affiliated organizations, or those of the publisher, the editors and the reviewers. Any product that may be evaluated in this article, or claim that may be made by its manufacturer, is not guaranteed or endorsed by the publisher.

## Footnotes

1
http://planttfdb.gao-lab.org/
