# Supplementary material for: Architecture and Dynamics of the Wounding-Induced Gene Regulatory Network During the Oolong Tea Manufacturing Process (Camellia sinensis)
Source: Front Plant Sci. 2022 Jan 27;12:788469. doi: 10.3389/fpls.2021.788469 (PMC8829136; doi:10.3389/fpls.2021.788469)
Supplement: Supplementary file 1 [file Data_Sheet_1.PDF]

Title: Architecture and dynamics of the wounding-induced gene regulatory network during the oolong tea manufacturing process (Camellia sinensis)

Authors: Yucheng Zheng[a], Qingcai Hu[a], Yun Yang[a], Zongjie Wu[a], Liangyu Wu[a], Pengjie Wang[a,b], Huili Deng[a], Naixing Ye[a,]*, Yun Sun[a,]*

Affiliation:

[a] Key Laboratory of Tea Science, College of Horticulture, Fujian Agriculture and Forestry University, Fuzhou 350002, China

[b] Shenzhen Branch, Guangdong Laboratory for Lingnan Modern Agriculture, Genome Analysis Laboratory of the Ministry of Agriculture, Agricultural Genomics Institute at Shenzhen, Chinese Academy of Agricultural Sciences, Shenzhen, 518120, China

*** Corresponding authors: Naixing Ye and Yun Sun *Email address*: ynxtea@126.com (N.Y.); sunyun1125@126.com (Y.S.)**

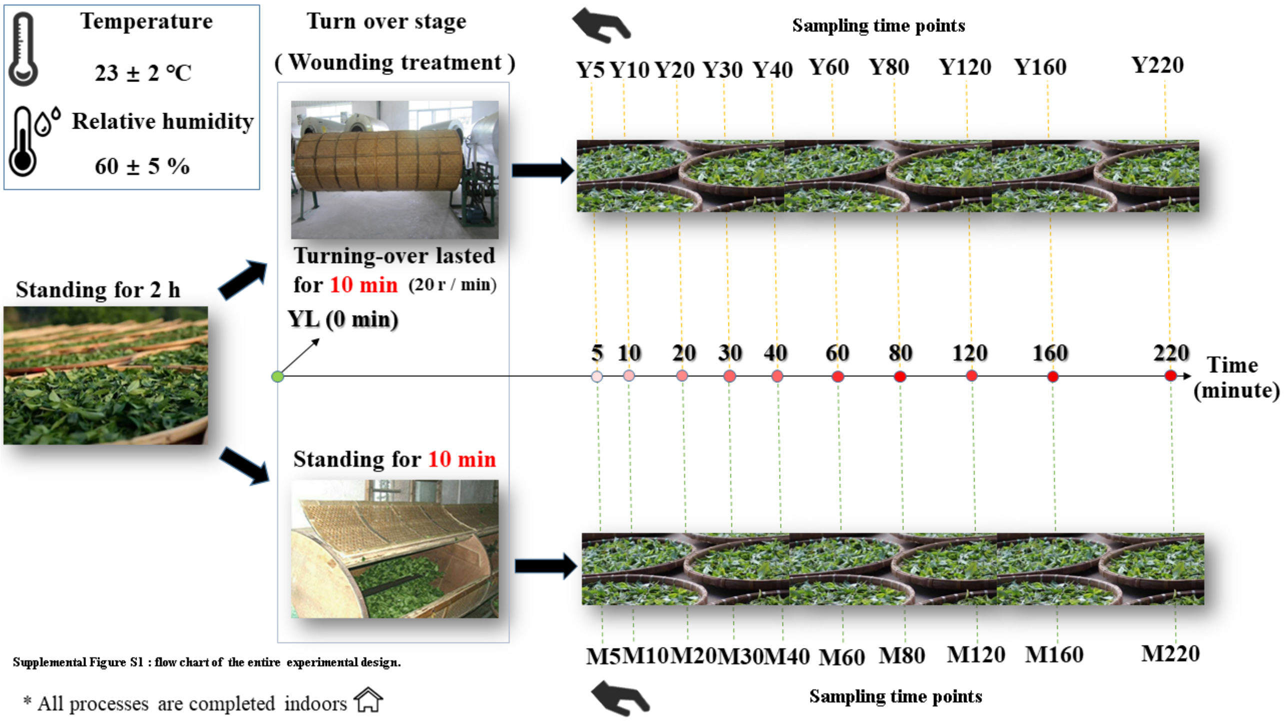

Supplemental Figure S1 flow chart of the entire experimental design. Red dots represents sampling time points.

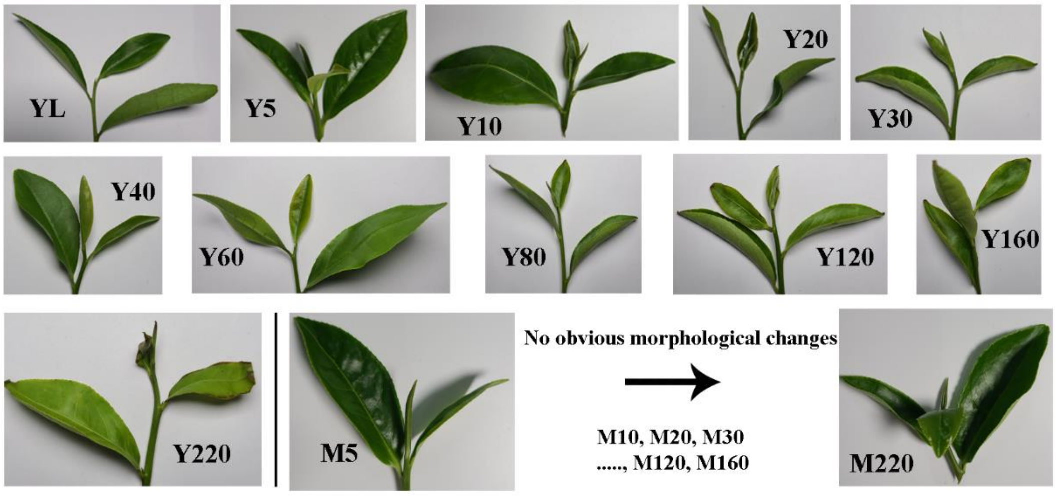

Supplemental Figure S2 sample photos of each time point.

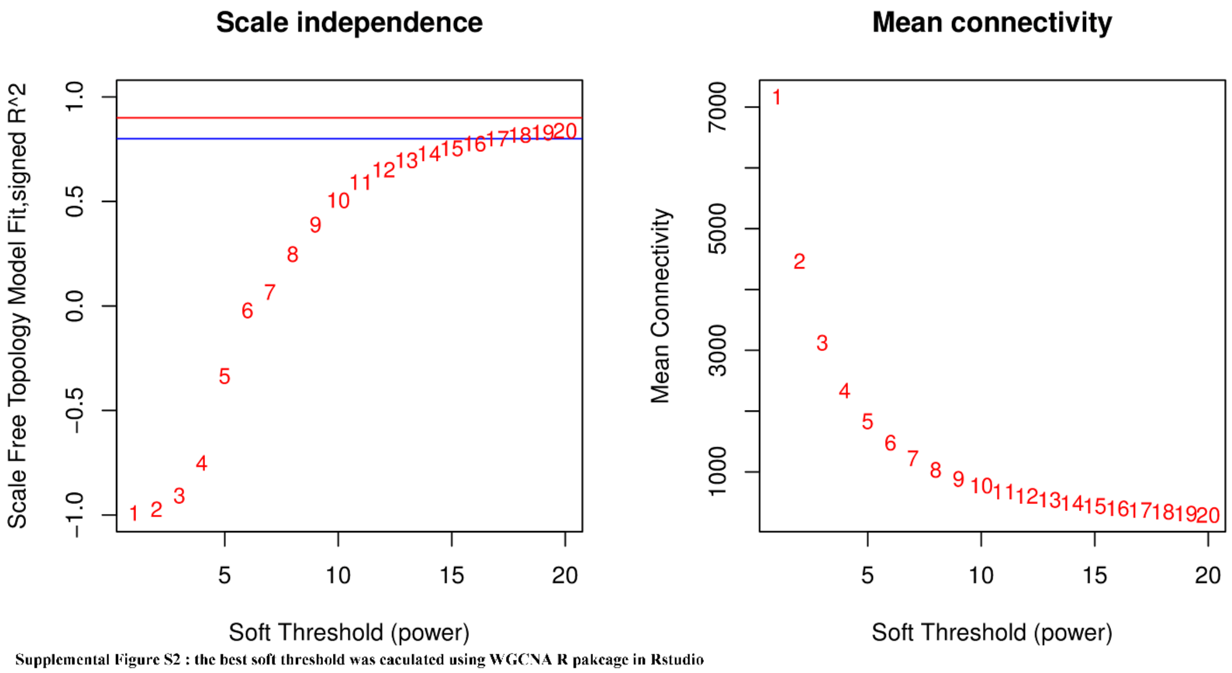

Supplemental Figure S3 the best soft threshold was calculated using WGCNA R package**.**

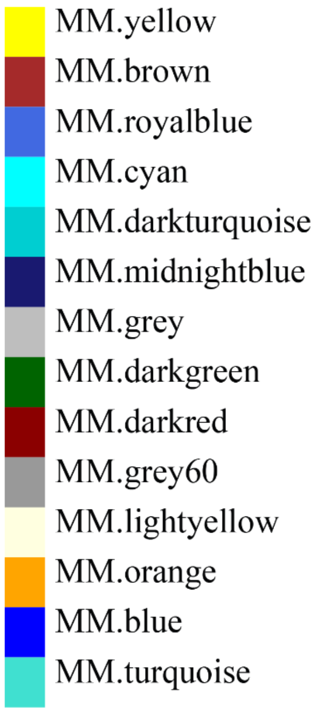

Supplemental Figure S4 the color information of different modules.

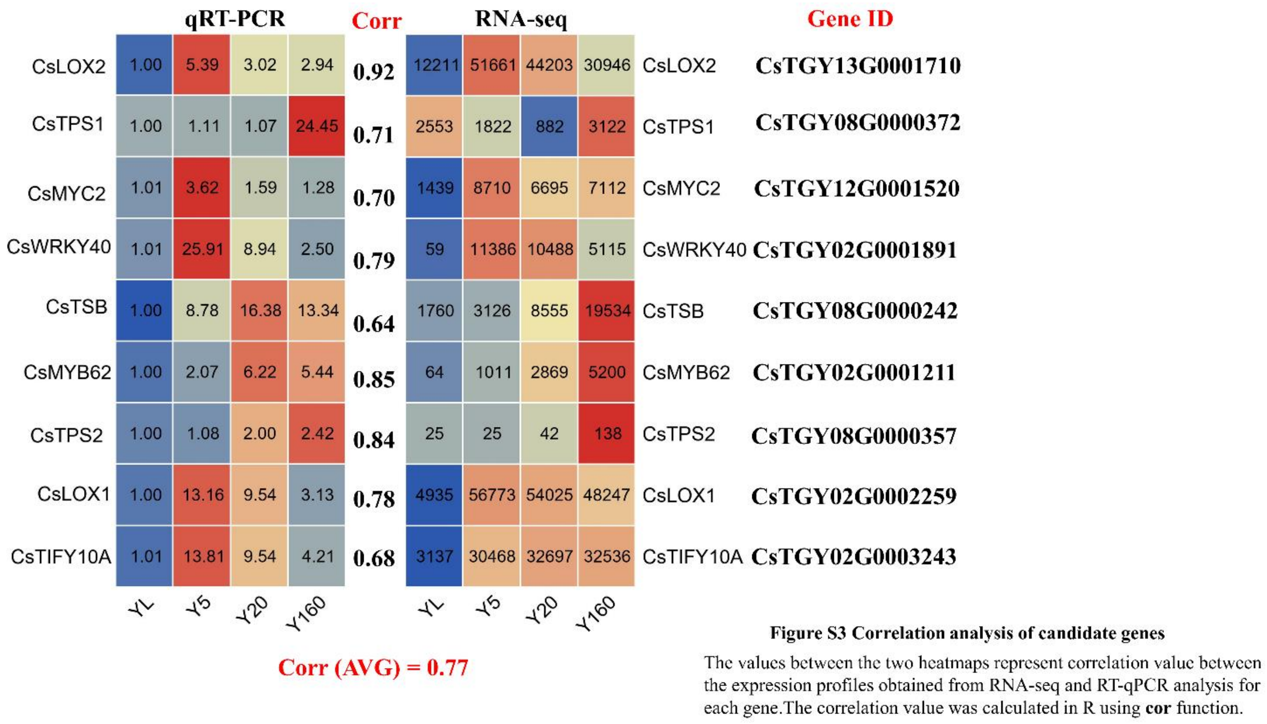

Supplemental Figure S5 Correlation analysis of candidate genes. The values between the two heatmaps represent correlation value between the expression profiles obtained from RNA-seq and RT-qPCR analysis for each gene. The correlation value was calculated in R using cor function.

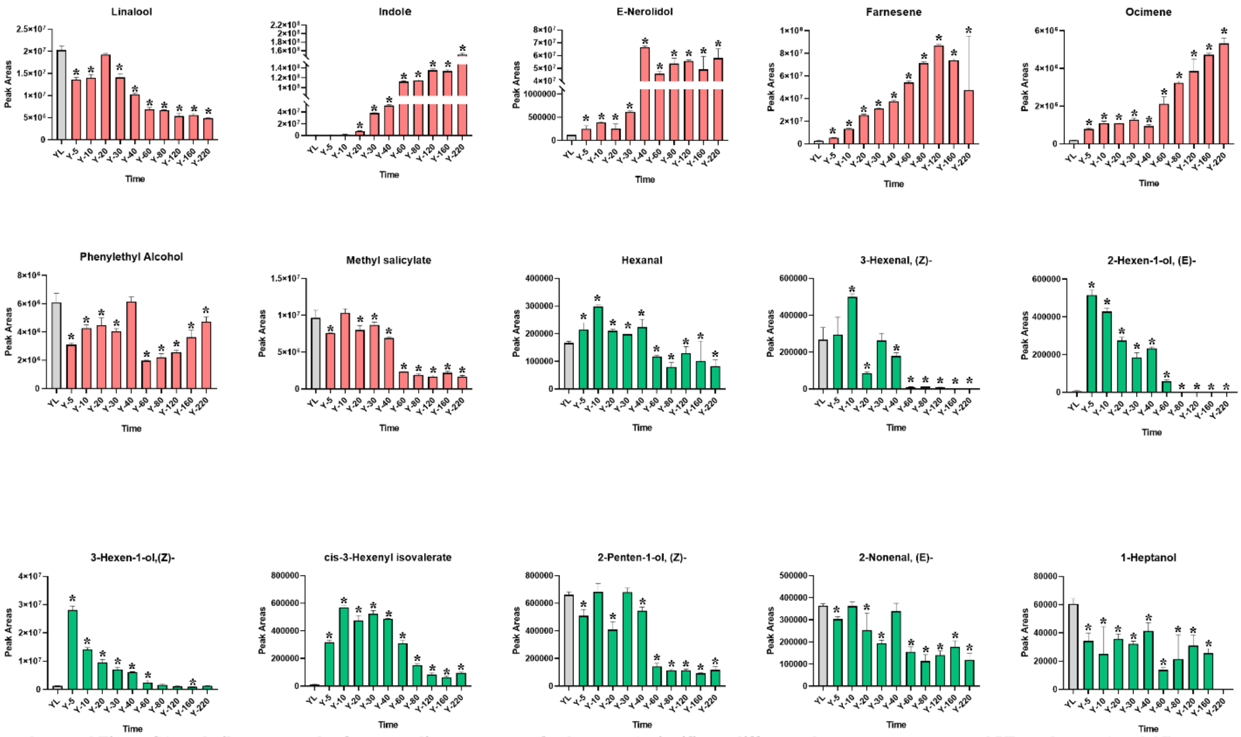

Supplemental Figure S4 : volatile compounds after wounding treatment of oolong tea. *, significant difference between treatments and YL tea leaves. (p < 0.05).

Supplemental Figure S6 volatile compounds after wounding treatment of oolong tea. *, significant difference between treatments and YL tea leaves ($p<0.05$).

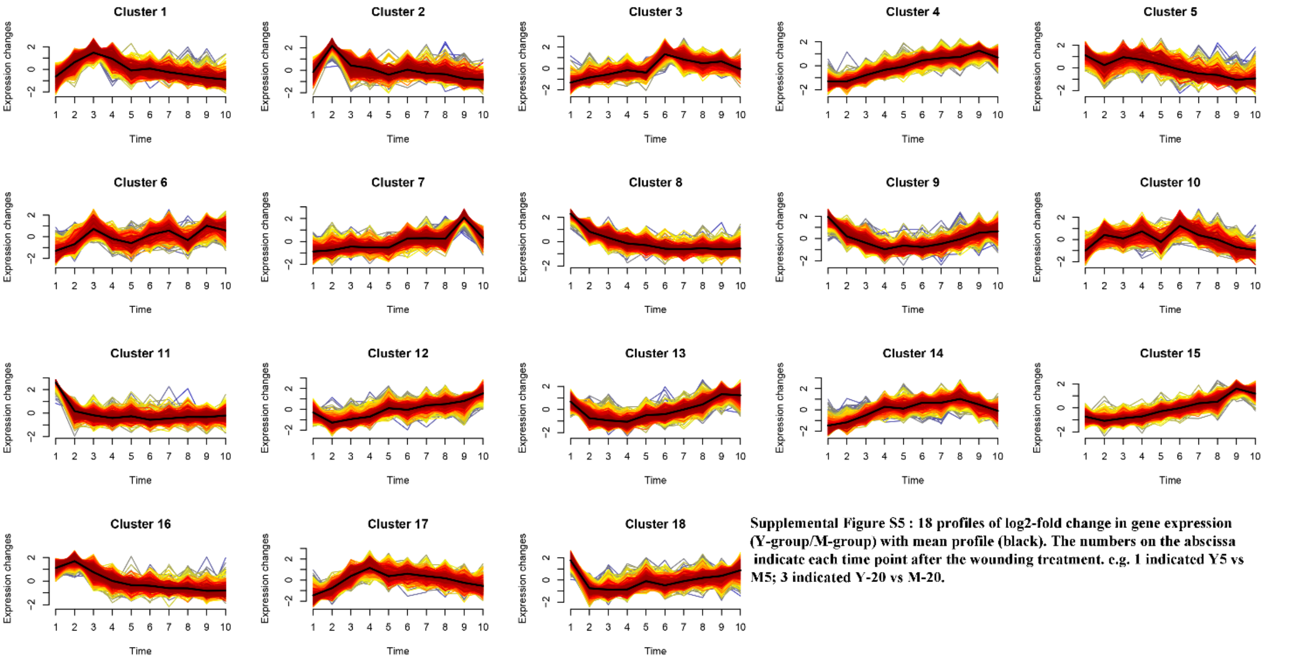

Supplemental Figure S7 18 profiles of log2-fold changes in gene expression (Y-group/M-group) with mean profile (Black line). The numbers on the abscissa indicate each time point after wounding treatment. e.g. 1 indicated Y5 Vs M5; 3 indicated Y-20 VS M-20.

Supplemental Figure S8 Total ion current of volatile metabolites profiling. Horizontal axis indicates retention time; Vertical axis indicates peak area. The same below.

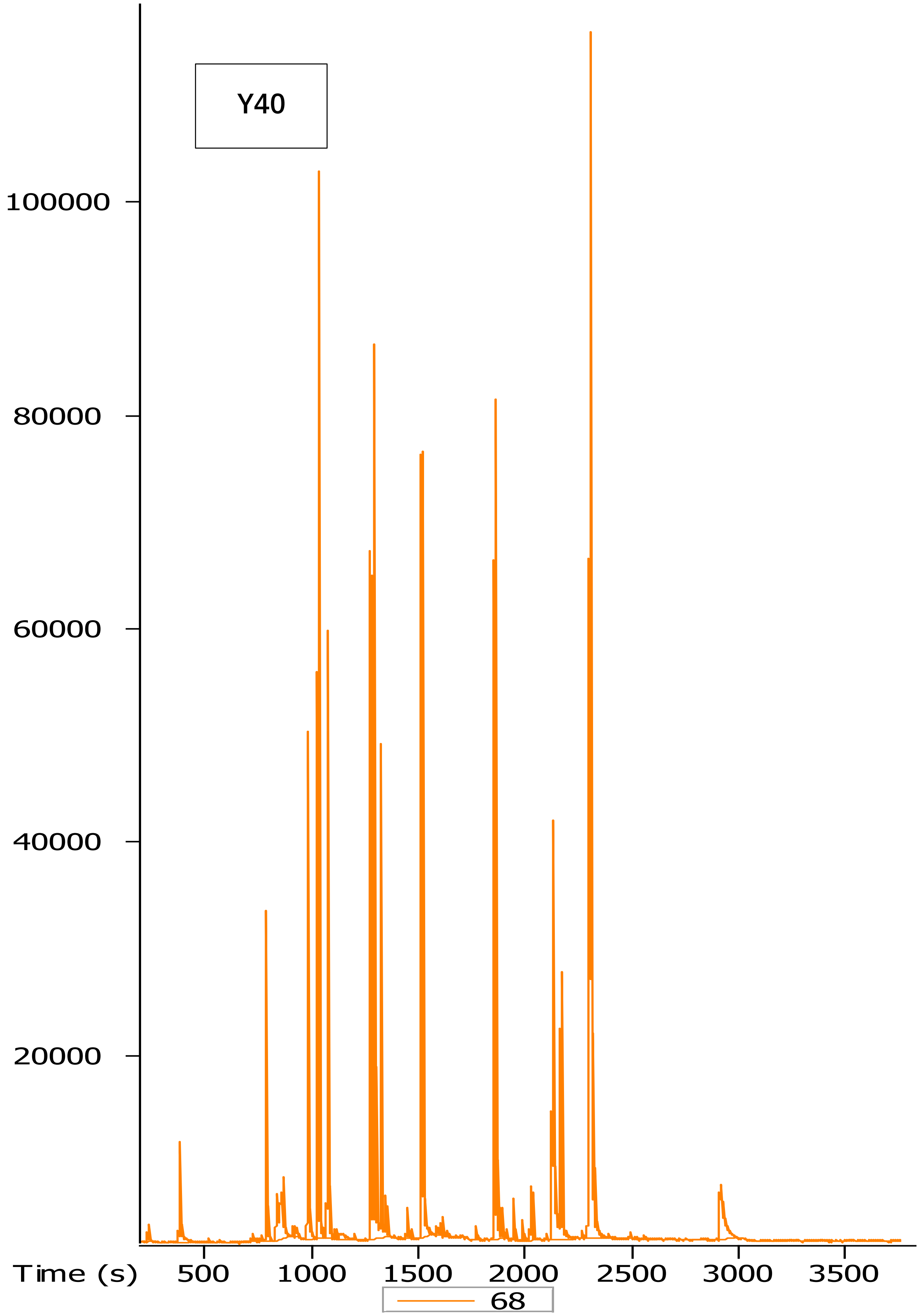

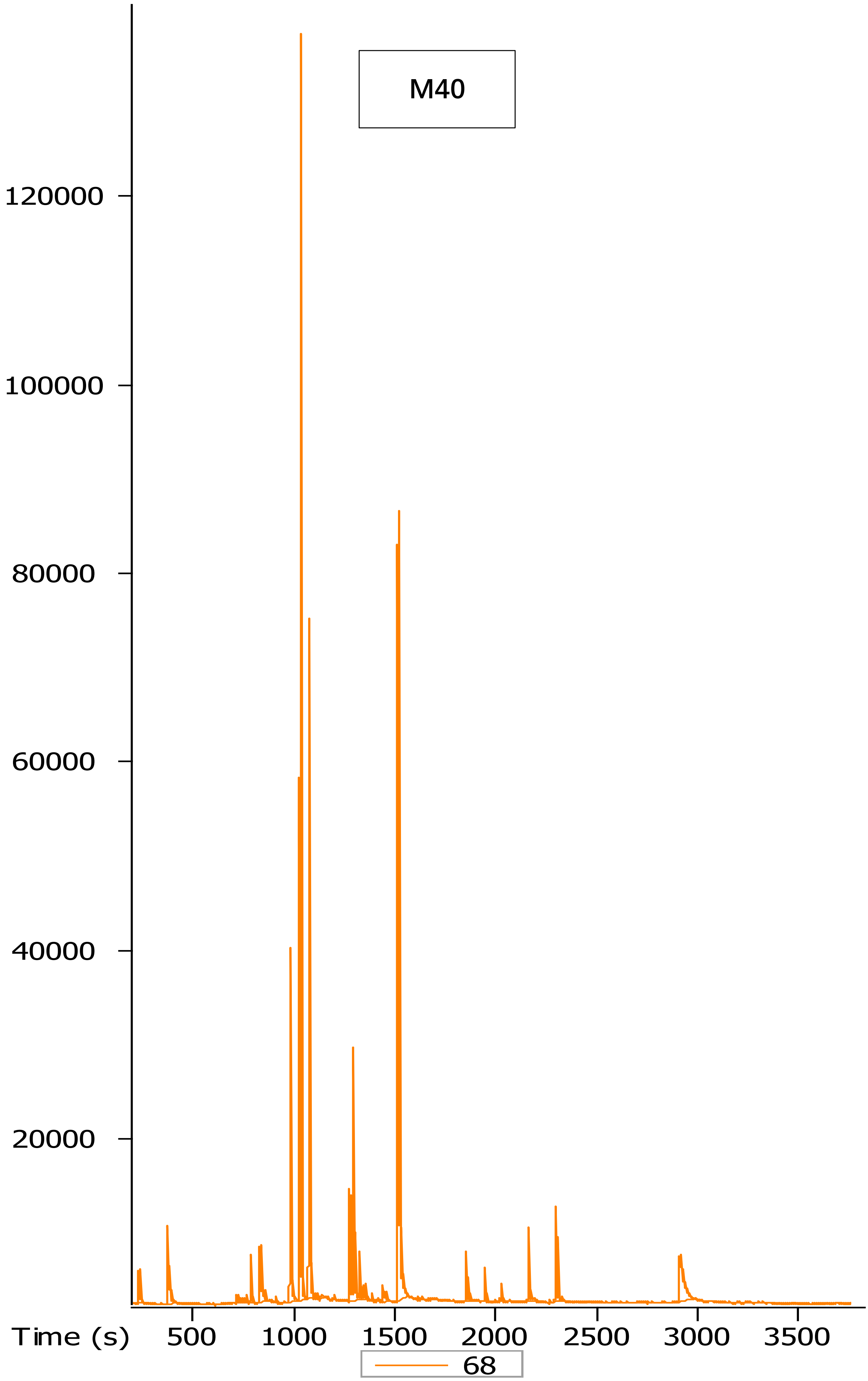
M40
120000
100000
80000
60000
40000
20000
Time (s)
500
1000
1500
2000
2500
3000
3500
68

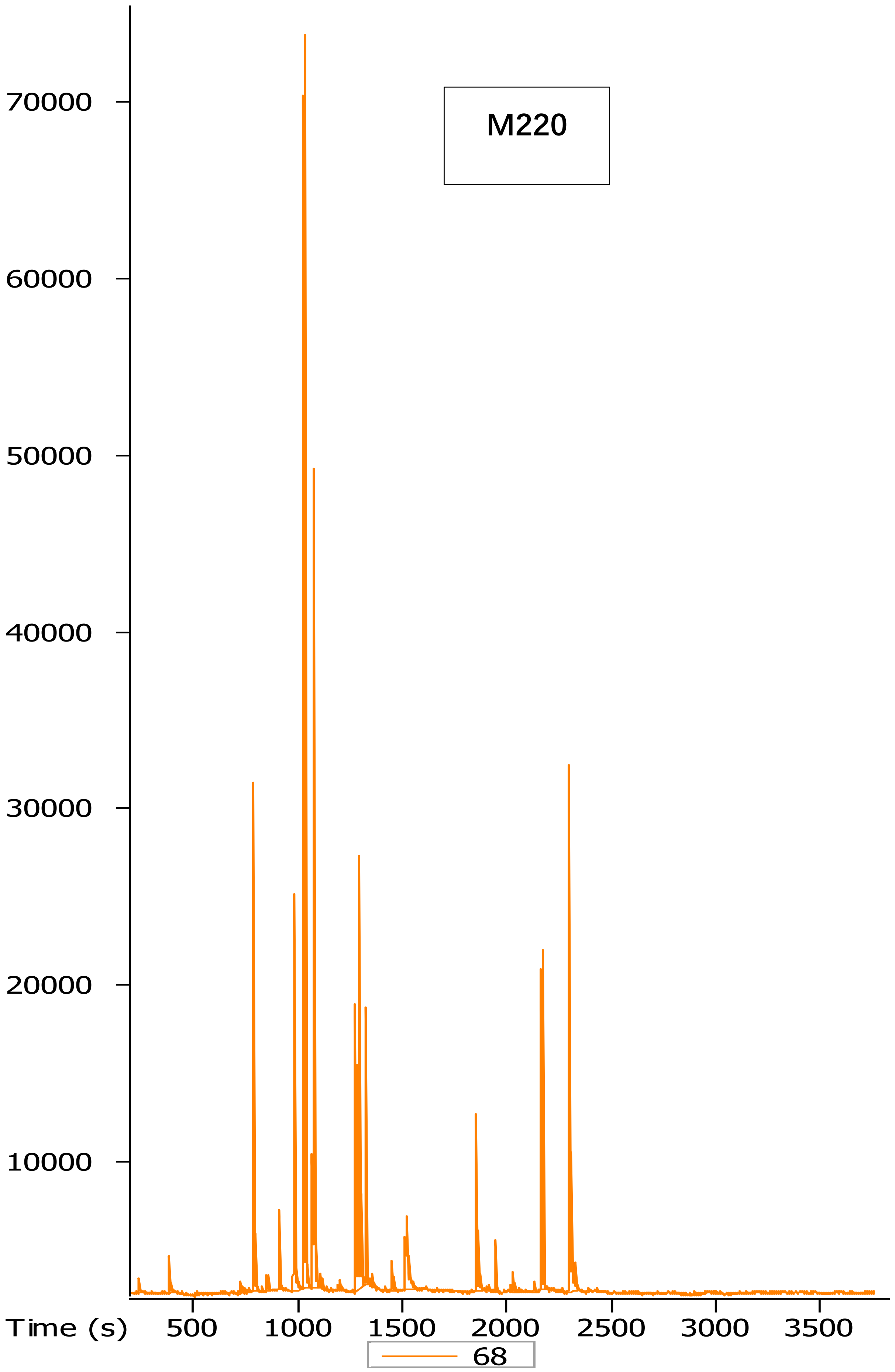

M220
70000
60000
50000
40000
30000
20000
10000
Time (s)
500
1000
1500
2000
2500
3000
3500
68

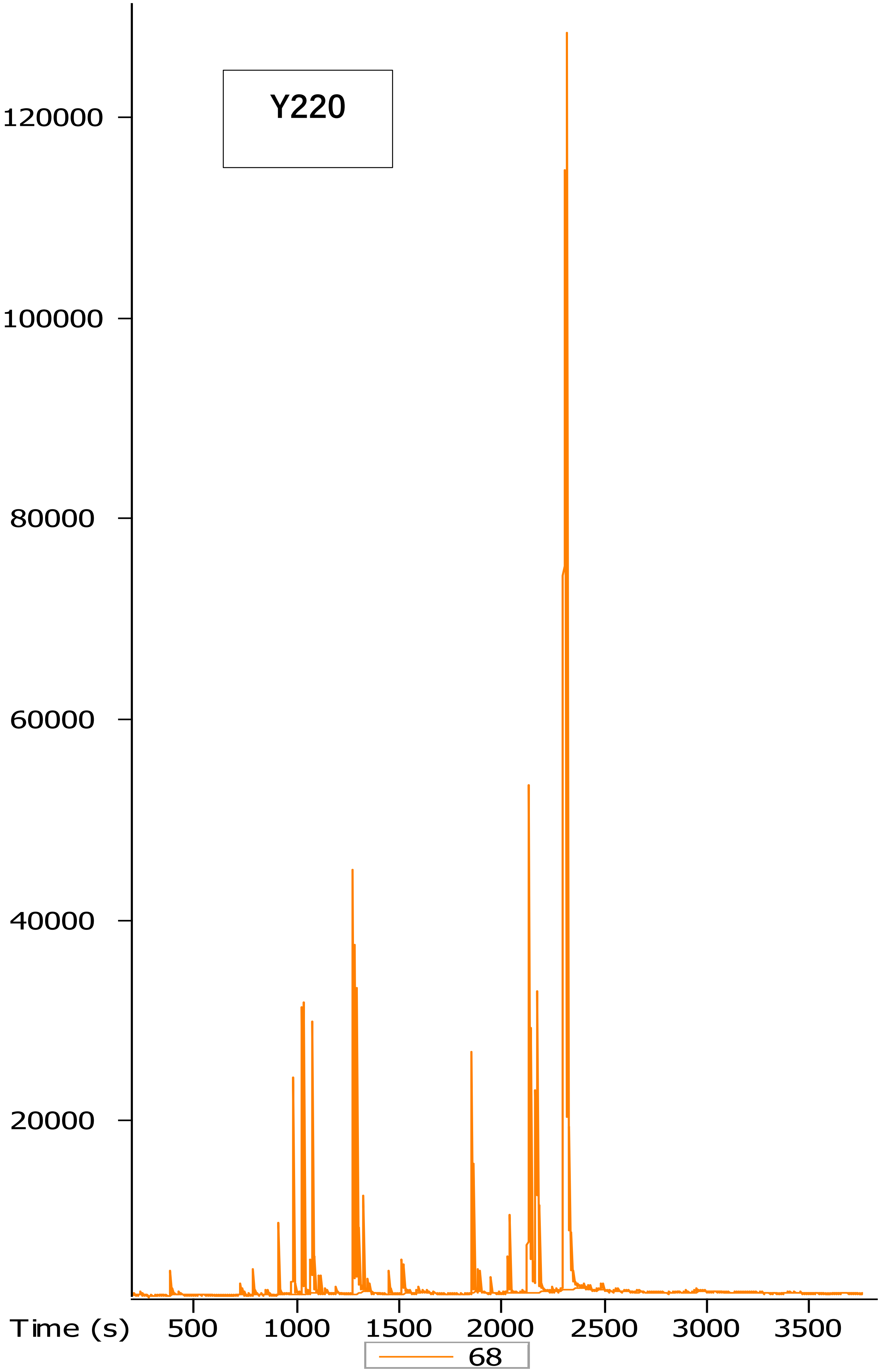

Y220
120000
100000
80000
60000
40000
20000
Time (s)
500
1000
1500
2000
2500
3000
3500
68

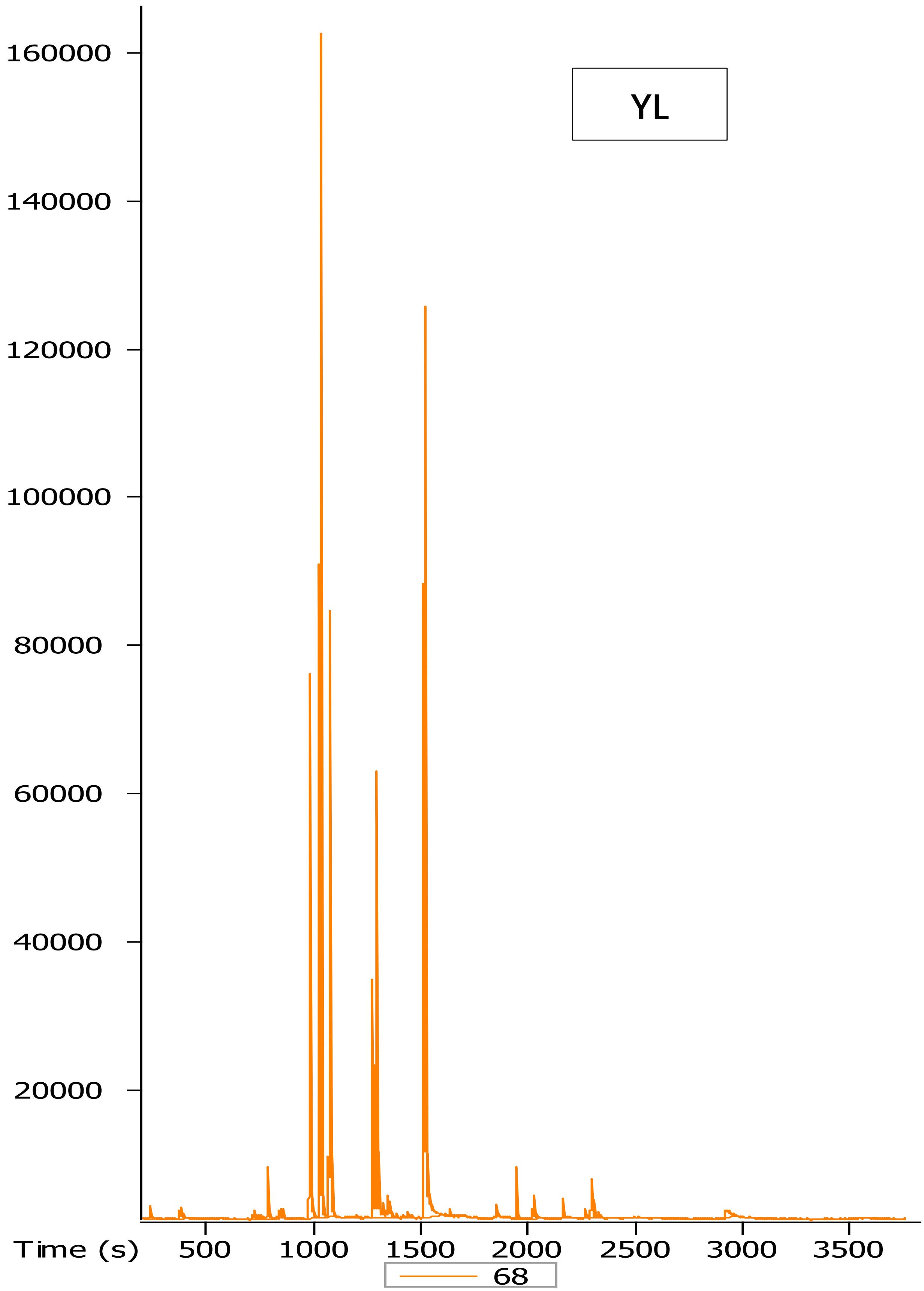

YL
160000
140000
120000
100000
80000
60000
40000
20000
Time (s)
500
1000
1500
2000
2500
3000
3500
68
